# Disturbed ***α***-Cell Function in Mice with ***β***-Cell Specific Overexpression of Human Islet Amyloid Polypeptide

**DOI:** 10.1155/2008/304513

**Published:** 2008-07-07

**Authors:** Bo Ahrén, Maria Sörhede Winzell

**Affiliations:** Department of Clinical Sciences, Lund University, 22184 Lund, Sweden

## Abstract

Exogenous administration of islet amyloid polypeptide (IAPP) has been shown to inhibit both insulin and glucagon secretion. This study examined *α*-cell function in mice with *β*-cell specific overexpression of human IAPP (hIAPP) after an oral protein gavage (75 mg whey protein/mouse). Baseline glucagon levels were higher in transgenic mice (41 ± 4.0 pg/mL, *n* = 6) than in wildtype animals (19 ± 5.1 pg/mL, *n* = 5, *P* = .015). In contrast, the glucagon response to protein was impaired in transgenic animals (21 ± 2.7 pg/mL in transgenic mice versus 38 ± 5.7 pg/mL in wildtype mice at 15 minutes; *P* = .027). Baseline insulin levels did not differ between the groups, while the insulin response, as the glucagon response, was impaired after protein challenge (*P* = .018). Glucose levels were not different between the groups and did not change significantly after protein gavage. Acetaminophen was given through gavage to the animals (2 mg/mouse) to estimate gastric emptying. The plasma acetaminophen profile was similar in the two groups of mice. We conclude that disturbances in glucagon secretion exist in mice with *β*-cell specific overexpression of human IAPP, which are not secondary to changes in gastric emptying. The reduced glucagon response to protein challenge may reflect a direct inhibitory influence of hIAPP on glucagon secretion.

## 1. INTRODUCTION

Islet amyloid polypeptide is a 37-amino-acid peptide, which is produced in the *β*-cells in the pancreatic islets [1–3]. It is coreleased with insulin [4], and exogenous administration of IAPP inhibits insulin secretion [2, 3, 5–7]. Several studies have also shown that exogenous administration of IAPP at supraphysiological doses inhibits glucagon secretion [8–11]. IAPP of the human form may lead to fibril formation, which causes amyloid deposition in the islets resulting in *β*-cell dysfunction and diabetes [12–14]. We have previously shown that mice with *β*-cell specific overexpression of the human form of IAPP (hIAPP) have defective insulin secretion and disturbed islet topography with centrally located glucagon producing *α*-cells [15, 16]. Whether these mice in addition have disturbed glucagon secretion is, however, not known. Therefore, the aim of the present study was to examine the glucagon response to an oral protein load in these mice compared to wildtype mice. Since IAPP has been shown to inhibit gastric emptying [11], also the gastric emptying rate in the transgenic and wildtype mice was evaluated with the previously described acetaminophen-test [17], to control for any differences in gastric emptying between the groups.

## 2. METHODS

### 2.1. Animals

Hemizygous transgenic mice with islet *β*-cell expression of hIAPP on a C57BL/6J/6xDBA/2 background were generated as previously described [18]. Transgenic status was determined by PCR using oligonucleotide primers directed against the hIAPP transgene [19]. The transgenic mice and their wildtype controls were kind gifts of Dr Steven E Kahn, University of Washington, Seattle, Wash, USA. Transgenic and wildtype mice were transported from the animal facility of the University of Washington, Seattle, to the In Vivo Department, Biomedical Center, Lund University, Lund, Sweden, after embryo transfer performed at Taconic A/S, Ry, Denmark. The animals were cross-bred for >16 generations to C57BL/6J mice. The animals were kept in a 12-hour light schedule (lights on at 0600 am) and given a standard pellet diet (fat 11.4%, carbohydrate 62.8%, protein 25.8% on an energy base, total energy 12.6 kJ/g) and tap water ad libitum. The Ethics Committee in Lund/Malmö approved the study.

### 2.2. Experiments

Following a four-hour period after removal of food from the cage, female transgenic and wildtype animals were anesthetized with an intraperitoneal injection of midazolam (Dormicum, Hoffman-La-Roche, Basel, Switzerland, 0.2 mg/mouse) as well as a combination of fluanison (0.4 mg/mouse) and fentanyl (0.02 mg/mouse; Hypnorm, Janssen, Beerse, Belgium). Thirty minutes later, a blood sample was taken from the retrobulbar, intraorbital, capillary plexus in heparinized tubes. Then, whey protein (100% Anywhey, 75 mg, Optimum Nutrition, Lindesberg, Sweden) and acetaminophen (paracetamol; Sigma Chemical Co, St Louis, Mo, 2 mg) dissolved in saline (total volume 500 *μ*L) were administered through a gastric tube (outer diameter 1.2 mm). After 15, 30, 60, and 120 minutes, blood samples, 75 *μ*L each, were collected. Blood was kept in heparinized tubes containing 5 *μ*L Trasylol (aprotinin; 10000 KIE/mL; Bayer HealthCare AG, Leverkusen, Germany), immediately centrifuged whereupon plasma was separated and stored at −20°C until analysis for glucose, glucagon, insulin, and acetaminophen.

### 2.3. Analyses

Plasma glucagon was determined with radioimmunoassay (Linco Res, St Charles, Mo, USA) with a guinea pig antiglucagon antibody, radioiodine labelled glucagon as tracer and glucagon standard. CV of the assay is 8% and the sensitivity of the assay is 10 pg/mL. The antibodies do not cross-react with GLP-1. Plasma insulin was determined with radioimmunoassay (Linco) with a guinea pig antirat insulin antibody, radioiodine labelled human insulin as tracer and rat insulin as standard. Plasma acetaminophen was determined with a colorimetric assay (Cambridge Life Science, Ely, Cambridgeshire, UK). Plasma glucose was determined with the glucose oxidase method.

### 2.4. Calculations and statistics

Means ± SEM are shown. Statistical comparisons were performed with the Student's *t*-test. For estimation of glucagon secretion, the increase in plasma glucagon levels during the first 15 minutes after protein gavage was estimated by subtracting baseline glucagon values from the 15-minute glucagon values. The area under the glucagon and insulin curves (AUCs) were also calculated using the trapezoid rule.

## 3. RESULTS

### 3.1. Glucagon response to oral protein

**Figure 1:**
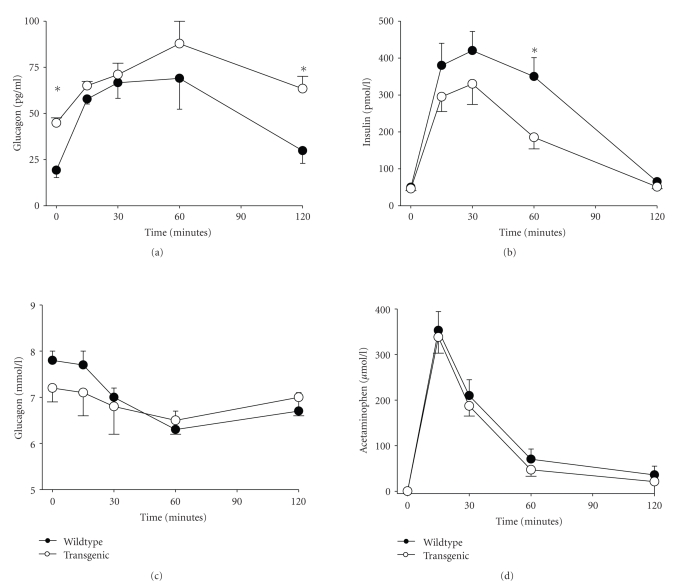
Plasma levels of glucagon, insulin, glucose, and acetaminophen following administration of whey protein (75 mg) and acetaminophen (2 mg) in female wildtype mice (*n* = 5) and transgenic mice with *β*-cell specific overexpression of hIAPP (*n* = 6). Means ± SEM are shown. Asterisks indicate probability level of random difference between the two groups (*P < .05).

Figure 1 (upper left panel) shows plasma glucagon levels during the oral protein challenge. Baseline glucagon levels were higher in the transgenic animals (41 ± 4.0 pg/mL, *n* = 6) than in the wildtype animals (19 ± 5.1 pg/mL, *n* = 5, *P* = .015). Glucagon levels at 15, 30, and 60 minutes after protein administration did not differ significantly between the groups, whereas the levels after 120 minutes were, again, significantly, higher in the transgenic animals (*P* = .008). Glucagon secretion was estimated as the change in glucagon levels during the first 15 minutes after protein gavage. This 15-minute glucagon response to protein administration was impaired in the transgenic animals, being 21 ± 2.7 pg/mL in transgenic mice versus 38 ± 5.7 pg/mL in wildtype mice (*P* = .027). The suprapasal AUC for glucagon for the entire 120-minute study period did not differ significantly between the groups, being 4.3 ± 1.1 *μ*g/mL × 120 minutes in wildtype mice versus 3.9 ± 0.9 *μ*g/mL × 120 minutes in transgenic mice.

### 3.2. Insulin and glucose responses to oral protein

Baseline insulin levels were 50 ± 5.1 påmol/L in wildtype animals and 46 ± 4.9 pmol/L in transgenic mice (NS). The insulin response to protein ingestion was impaired in transgenic mice; the suprabasal AUC for insulin for the 120-minute study period was 27.2 ± 3.1 nmol/L × 120 minutes in wildtype animals versus 16.5 ± 3.9 nmol/L × 120 minutes in transgenic animals (*P* = .018) (Figure 1, upper right). Baseline glucose levels were 7.2 ± 0.3 mmol/L in wildtype animals and 7.8 ± 0.2 mmol/L in transgenic mice (NS); glucose levels did not change significantly during the test (Figure 1, lower left panel).

### 3.3. Acetaminophen response to acetaminophen administration

Figure 1 (lower right) shows the acetaminophen concentrations during test. Plasma acetaminophen increased to a maximum level at 15 minutes after administration, thereafter it gradually fell. There was no significant difference between the groups in plasma acetaminophen.

## 4. DISCUSSION

This study evaluated the islet hormone responses to oral protein ingestion in mice with *β*-cell specific overexpression of human IAPP. It was found that the insulin response to protein was impaired in transgenic mice. This confirms that these mice have impaired insulin secretion, as previously was reported also after oral glucose challenge [15]. The main novel finding in this report is, however, that the transgenic mice have also changes in the glucagon levels. Thus, the mice were found to have higher baseline glucagon levels than their wildtype counterparts and yet they have a reduced glucagon response to protein administration. The mechanism of the high-baseline glucagon remains to be established. It is, however, consistent with the disturbance in islet topography in these mice. Thus, we have previously shown that the islets of these mice have enlarged population of glucagon producing *α* cells as opposed to the reduced *β*-cell immunostaining in these animals which is associated with significantly reduced islet insulin content [16]. This hyperglucagonemia may be the result of the reduced islet insulin, in view of the inhibitory influence of insulin on glucagon secretion. At the same time, the glucagon response to the protein administration was impaired, which may be explained by the transgene, because IAPP is known to inhibit glucagon secretion [8–11]. Hence, high-baseline glucagon and impaired glucagon response to stimulation are two characteristics of the hIAPP transgene, and may have different mechanisms.

In this study, we also determined the acetaminophen concentration after acetaminophen administration to determine whether gastric emptying had been altered in the transgenic mice. Previously, inhibition by IAPP of gastric emptying has been demonstrated [11] and changes in gastric emptying would be a mechanism for changes in glucagon secretion after protein administration. The acetaminophen test has previously been validated in humans [20] and used in a previous study in mice [17]. It is based on the poor absorption of acetaminophen from the stomach and the rapid and almost complete absorption from the small intestine. This implies that plasma acetaminophen profiles give an estimation of gastric emptying, which also has been verified as good correlation with isotopic technique measurements of gastric emptying [21, 22]. We found that there was no difference in plasma acetaminophen profiles between transgenic and wildtype mice. This shows that the increase in *β*-cell IAPP expression does not affect gastric emptying, and, therefore, the inhibited glucagon response to oral protein in these mice is not due to impaired gastric emptying.

In conclusion, this study has shown that *β*-cell specific overexpression of human IAPP increases baseline glucagon levels and impairs the glucagon response to oral protein in association with impaired insulin response. This shows that a disturbed *α*-cell function in these mice is evident in association with the previously described disturbed *β*-cell function [15, 16].
